# Repeat mild traumatic brain injuries (RmTBI) modify nociception and disrupt orexinergic connectivity within the descending pain pathway

**DOI:** 10.1186/s10194-023-01608-y

**Published:** 2023-06-14

**Authors:** Jennaya Christensen, Naomi MacPherson, Crystal Li, Glenn R. Yamakawa, Richelle Mychasiuk

**Affiliations:** Department of Neuroscience, Central Clinical School, 99 Commercial Road, VIC 3004 Melbourne, Australia

**Keywords:** Concussion, CGRP, Lateral hypothalamus, Lateral parabrachial nucleus, Periaqueductal grey, Post-traumatic headache

## Abstract

Repeat mild traumatic brain injuries (RmTBI) result in substantial burden to the public health system given their association with chronic post-injury pathologies, such as chronic pain and post-traumatic headache. Although this may relate to dysfunctional descending pain modulation (DPM), it is uncertain what mechanisms drive changes within this pathway. One possibility is altered orexinergic system functioning, as orexin is a potent anti-nociceptive neuromodulator. Orexin is exclusively produced by the lateral hypothalamus (LH) and receives excitatory innervation from the lateral parabrachial nucleus (lPBN). Therefore, we used neuronal tract-tracing to investigate the relationship between RmTBI and connectivity between lPBN and the LH, as well as orexinergic projections to a key site within the DPM, the periaqueductal gray (PAG). Prior to injury induction, retrograde and anterograde tract-tracing surgery was performed on 70 young-adult male Sprague Dawley rats, targeting the lPBN and PAG. Rodents were then randomly assigned to receive RmTBIs or sham injuries before undergoing testing for anxiety-like behaviour and nociceptive sensitivity. Immunohistochemical analysis identified distinct and co-localized orexin and tract-tracing cell bodies and projections within the LH. The RmTBI group exhibited altered nociception and reduced anxiety as well as a loss of orexin cell bodies and a reduction of hypothalamic projections to the ventrolateral nucleus of the PAG. However, there was no significant effect of injury on neuronal connectivity between the lPBN and orexinergic cell bodies within the LH. Our identification of structural losses and the resulting physiological changes in the orexinergic system following RmTBI begins to clarify acute post-injury mechanistic changes that drive may drive the development of post-traumatic headache and the chronification of pain.

## Introduction

Although concussions / mild traumatic brain injuries (mTBI) are often a one-off occurrence, a subset of the population experience repeat mTBIs (RmTBI). The World Health Organization estimates that the prevalence of mTBI is between 100–300/100,000 people per year, however, the hypothesised true population prevalence is likely much higher due to underreporting [1]. While there is currently no research specifying the true population risk of incurring RmTBIs [2], specific populations, such as military personnel, athletes, and victims of domestic violence, are known to be at higher risk [2, 3]. For the general public, identified risk factors for RmTBI include male gender, alcohol intoxication, and a prior mTBI [3].

Chronic symptomatology following RmTBIs is costly, as disability prevalence is estimated at 47%, with approximately 20% of individuals exhibiting a severe degree of disability [4]. One of the commonly reported physiological outcomes post-RmTBI is chronic pain. General incidence of chronic pain post-mTBI has been estimated between 58% and 75.3% [5, 6]. The most common chronic pain sequelae associated with RmTBI is chronic post-traumatic headache (CPTH) [5–7], which has prevalence rates as high as 89% [8]. Although often ineffective, chronic pain following mTBI has been treated similarly to chronic pain conditions that are not derived from a concussive injury. For example, given that CPTH is most commonly described to have migraine-like qualities or present like a tension-type headache [9, 10], trialled pharmaceutical management for a CPTH involves the standard treatment for migraine/tension headaches. The ineffectiveness of migraine therapy for CPTH suggests that CPTH is, in part, a different entity to primary headache phenotypes [10–12].

Research suggests that a dynamic interaction between neurobiological and physiological mechanisms drives the transition from acute to chronic pain post-mTBI [7]. Alterations to the cortical and sub-cortical processing of pain may result from a state of imbalanced facilitation and inhibition of pain mechanisms following RmTBIs [13–15]. Sub-cortical anatomical sites within the descending pain modulation (DPM) pathway, including the periaqueductal gray (PAG), are thought to be affected post-mTBI. These brain regions respond in an integrated means to higher order cortical input to reduce subjective pain experiences [16]. It is therefore plausible that traumatic causative mechanisms following RmTBI have a multi-tiered influence within the DPM pathway [17]. Given that dysfunctional signalling within this pathway could produce the interrelated changes necessary for the chronification of pain, there is a need to investigate signalling pathways that act within the DPM pathway and are vulnerable to the post-traumatic neuropathological changes associated with RmTBIs.

The orexinergic system, which innervates the DPM pathway and has an established role in the pathogenesis and recovery from multiple neurological disorders [18], provides a possible neuronal signalling pathway that meets these criteria. The orexinergic system’s contribution to pain modulation is exclusively anti-nociceptive, which occurs supra-spinally and spinally [19]. Pain modulation can occur via direct influence of the orexinergic system or indirectly via modulation of other central nervous system (CNS) neurotransmitters, such as the serotonergic, histaminergic, noradrenergic, and dopaminergic systems [19, 20]. Within these neurotransmitter systems, both direct excitatory modulation and indirect inhibitory modulation occurs, which enables orexinergic influence to have dynamic flexibility [19, 20].

The lateral parabrachial nucleus (lPBN) in particular, plays a key role in orexinergic pain modulation. Glutamatergic in nature, lPBN neurons exhibit an excitatory effect over orexin neurons particularly within the hypothalamic dorsomedial nucleus and perifornical area [21, 22]. The influence of the lPBN in pain modulation is thought to occur directly through synaptic transmissions with orexin neurons in the lateral hypothalamus (LH), and indirectly by a prominent overlapping of the brainstem regions that both it and orexinergic neurons innervate [21]. The lPBN primarily integrates and transmits, with some modulatory function, nociceptive information to higher-order structures [23]. Uniquely, within the brainstem, lPBN output projections relay nociceptive information directly to higher structures, including the amygdala and hypothalamus, without first relaying through the thalamus [24]. To date, no literature has analysed the relationship between RmTBIs and the lPBN although it could be hypothesized that the traumatic insult of RmTBIs would alter orexinergic physiology secondary to microscopic hypothalamic damage and/or localised neuronal protective mechanisms. These concurrent neuropathological changes may generate a susceptibility to chronic pain and CPTH by diminishing the anti-nociceptive functioning of the orexinergic system. We therefore used a translationally relevant pre-clinical model to investigate the influence of RmTBIs on nociception and orexinergic neurons, with a specific focus on their connectivity with the lPBN and PAG.

## Methods

### Animal characteristics

All research was completed in alignment with the Precinct Animal Centre (PAC) Animal Care guidelines at Monash University. Ethics approval was obtained by the Alfred Medical Research and Education Precinct Animal Ethics Committee, with the care of the animals completed in accordance with the ethical guidelines stipulated for this project (E/1933/2019/M).

This research was carried out with male Sprague Dawley rats, approximately 8 weeks old (postnatal day (P) = 58)), weighing between 268 and 363 g at the start of the experiment. Seventy rats were obtained from the Monash Animal Research Platform. The animals were housed in groups of 2–3, with *ad libitum* access to food and water. The cages were kept within the PAC facility and were maintained on a 12 h/12hr light/dark cycle (lights on at 6:00am), and an average room temperature of 21 °C.

**Fig. 1:**
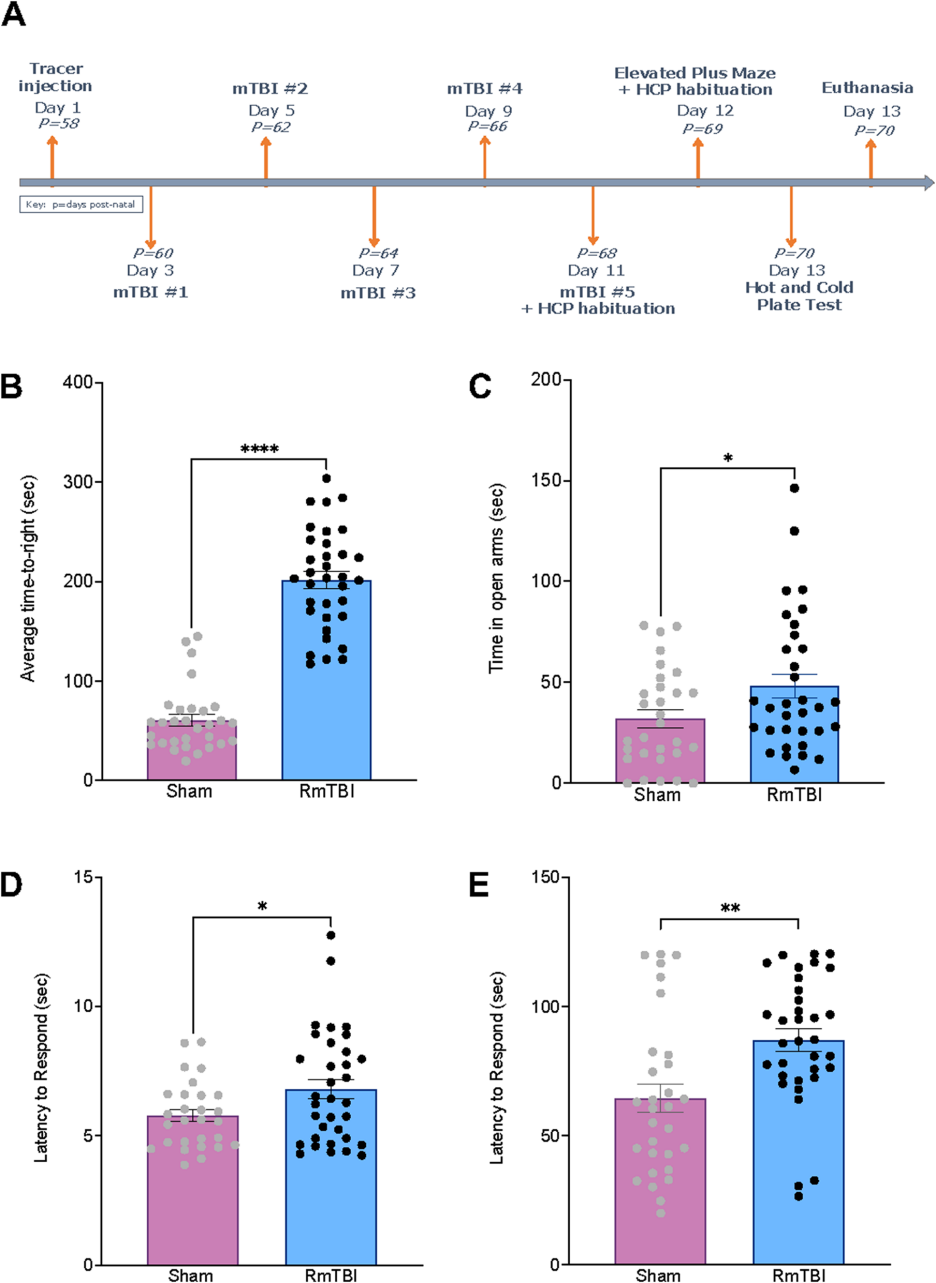
Study timeline and bar graphs displaying behavioural results. **A** Illustrative representation of study manipulations in relation to postnatal age; **B) **Average time-to-right following mTBI or sham injury; **C)** Total time spent in open arms of the EPM; **D)** Latency to react on the hot plate; and **E)** Latency to react on the cold plate. Mean ± SEM shown. * *p* < .05, ** *p* < .01, **** *p* < .001

The experimental research was completed over 7 cohorts, where *n* = 10 within each cohort. At P58, surgery was performed to inject a neuronal tract-tracer into specified areas of analysis for this research project; the lPBN or PAG. For each cohort, a particular anatomical site was assigned. To ensure that appropriate healing had occurred, the first mTBI was performed two days after surgery was completed. Five mTBIs were completed in total, with each being performed at two-day intervals. To test anxiety-like behaviour, the elevated plus maze (EPM) was completed 24 h after the last mTBI; post-injury day (PID) 1. Habituation to hot/cold plate (HCP) occurred over two days; PID0 and PID1. The HCP test was administered on PID2. Following completion of the HCP, the rats were euthanised. All procedures were carried out by researchers blinded to the experimental groups and conditions. See Fig. 1 for experimental timeline.

### Tracer iontophoresis

Before undergoing surgery, each rat was weighed and anesthetised using 5% isoflurane in a mix of 2 L/min of oxygen. Once non-responsive to a toe pinch, animals were placed on an absorbent covered heat pad and into a stereotaxic frame. While in the stereotaxic frame, isoflurane anaesthesia was maintained using a nose cone, which administered 2% isoflurane mixed with 1 L/min of oxygen. Each rat received pre-operative care that included: 3ml subcutaneous injection of saline, 0.05 mg/kg subcutaneous injection of buprenorphine, and lubricating PolyEye gel administration. Following incision through cranial soft tissue, the skin and subcutaneous tissues were reflected laterally. The Paxinos and Watson Rat Brain atlas [25] was used to guide the coordinates for tracer injection into lPBN and PAG. A burr hole was then drilled in the skull at these sites, with careful attention paid to avoid contact and disruption of the dura.

The anterograde tract tracer biotinylated dextran amine (BDA) was iontophoretically applied ipsilaterally into the lPBN at the following stereotaxic coordinates: 2.00 mm lateral to bregma, 9.40 mm posterior to bregma, and 6.00 mm ventral relative to dura. BDA was dissolved in sterile saline to create a 10% solution and 5µL was back filled into 30–50 μm glass micropipettes. The pipettes were lowered using a negative retaining current to minimize leakage along the tract. BDA was dispersed into brain tissue through a positive driving current of 5µA at pulsating 7 Hz (7 s on followed by 7 s off). This was administered for a total of 15 min, and then left in for a further five minutes before removal.

Ipsilateral tract-tracer injections of retrograde tract tracer, cholera toxin B subunit (CTB), into the ventrolateral PAG were delivered via iontophoresis at the following stereotaxic coordinates: 0.60 mm lateral to bregma, 7.08 mm posterior to bregma, and 5.40 mm ventral relative to the dura. CTB was dissolved in distilled water to create a 1% solution and 5µL was back-filled into 30–50 μm glass micropipettes. CTB was dispersed into brain tissue through a positive driving current of 1.5mA at pulsating 7 Hz (7 s on followed by 7 s off). This was administered for a total of 15 min, and then left in for a further five minutes before removal to facilitate diffusion.

Following the necessary time for ionic dispersion, the glass pipette was slowly retracted. Cranial tissue was then sutured together with 4/0 Monosyn absorbable sutures and the rats were placed in a recovery cage on a heated pad.

Five rats were lost across the 7 cohorts during surgery, leading to a final sample size of 65.

### Injury induction (RmTBIs)

For each cage, rats were randomly assigned to receive RmTBI or sham injuries. The five mTBIs were completed on P60, P62, P64, P66, and P68. A two-day inter-injury interval was chosen to enable observation of cumulative neuropathological effects of RmTBI, whilst still providing recovery time for the animals. The lateral impact model (LI), which generates acceleration/deceleration alongside rotational forces commonly seen in sports-related concussions and motor vehicle accidents [26], was used to induce the injuries.

For experimental validity, circadian timing of injury was kept consistent; with all injuries and sham procedures commencing at 9am. Before induction of sham or mTBI, all rats were anaesthetised. Rats were exposed to 5% isoflurane in a mix of 2/L min oxygen, for approximately two minutes. Loss of toe-pinch reflex was used to indicate an adequate loss of consciousness. Rats assigned to RmTBI were placed on a Teflon Board in a prone position. Their head was positioned against an aluminium protective helmet, which was situated directly opposite to the lateral impactor. A 50 g weight was then propelled towards the left temporal lobe, utilising pneumatic air pressure, at an average speed of 9.13 +/- 0.05 m/s (~ 92Gs). The impact induced a horizontal 180° rotation of the rat, resulting in acceleration and deceleration of the brain, along with a horizontal 180° rotation. The rats were then transferred to a recovery cage where their time-to-right was recorded. For rats assigned to a sham injury, placement within the lateral impact device was identical to those who underwent RmTBIs. However, no lateral impact was administered, so rats were removed without injury and placed in the clean recovery cage. Their time-to-right was also recorded.

Time-to-right is a commonly used behavioural assessment for loss of consciousness [26–29]. As soon as injury occurred, via lateral impact, or voiced to start for sham procedures, a stopwatch was started. Animals were then removed from the lateral impact device, and observation continued in the clean recovery cages where the animals were laid supine. Once animals regained muscle tone, as indicated by flipping prone and beginning ambulation, time recording stopped. The time that lapsed was deemed ‘time-to-right.’

### Behavioural testing

EPM – The EPM was used to measure anxiety-like behaviour [30]. The maze was comprised of black Plexiglass, shaped in a cross that was raised 51 cm off the ground. There were four arms, each measuring 51 × 11 cm, with two closed (high vertical walls on each side) and two open (no surrounding walls). Each rat was placed in the centre of the EPM with their heads facing an open arm. The animal was left in the EPM for 5 min and their behaviour was tracked with TopScan software using an overhead camera. Between each rat, the EPM was cleaned with 70% ethanol.

HCP - The HCP was performed on PID 2 and was used as a thermal indicator of nociceptive sensitivity, as indicated by Le Bar et al. [31] and Barrot et al. [32]. Prior to testing, animals were habituated to the device for two days. During habituation, animals were placed into the apparatus, which was left at room temperature, for two minutes before removal. For hot plate the temperature of the base plate was set to 52 °C. Each rat was then placed into the apparatus, with the latency measured as the time until the first behavioural indicator of a nociceptive response. The same protocol was employed for cold plate, however, the base plate was set to 2 °C. The apparatus was cleaned with Virkon between each rat.

### Tissue preparation and immunohistochemical processing

All rats were euthanised, on PID2. Following deep anaesthesia with 5% isoflurane in 2 L/min of oxygen, each rat received an intraperitoneal injection of 1ml (160 mg/ml) of pentobarbitone. Cardiac blood was then drawn. Next, the rats underwent a transcardial perfusion with phosphate-buffered saline (PBS) followed by 4% paraformaldehyde (PFA). Brains were extracted and post-fixed in 4% PFA for 24 h, at 4 °C. After the initial 24 h, the brains were switched to a 30% sucrose solution. Blood was centrifuged for 20 min at 4 °C to enable serum collection. Serum was then stored at -80 °C until needed for analysis.

Rat brains were sliced coronally via a cryostat, at a thickness of 20 μm. Regions of interest were collected at a rate of one in two sections and mounted onto Superfrost plus slides. To ensure regions were correctly identified, anatomical features visualised were correlated to those in Paxinos and Watson Rat Brain atlas [25].

All immunohistological staining began with sections undergoing three stages of washing in 0.3% Triton-X PBS (TX-PBS). For anterograde tract-tracing brains, slides were then blocked with a solution of normal goat serum (NGS) and TX-PBS for 1 h. Following this, they were treated with a solution of NGS, TX-PBS, and specific primary antibodies, for 1 h at room temperature. For slides corresponding to the LH, this included a rabbit anti-Orexin-A antibody (1:500; Abcam ab255294), while lPBN slides did not require a primary antibody. Slides were then incubated in these primary antibodies overnight (~ 18 h) at 4 °C. The following day slides were incubated, at room temperature, on a shaking platform for one hour. Three TX-PBS washes, for a total of 30 min, was then completed. Next, slides were in fluorescent secondaries for one hour, at a dilution rate of 1:200. For both LH and lPBN slides this involved a solution of TX-PBS, goat anti-rabbit Alexa Fluor 647 (ThermoFisher #A-21,245), Streptavidin Alexa Fluor 488 (ThermoFisher S32354) and Hoechst 33,342. Following this stage, slides were protected from light exposure. After the incubation of fluorescent secondaries, the slides were washed with TX-PBS for 10 min, which was repeated three times.

For retrograde tract-tracing brains, slides were blocked with a solution of NGS and TX-PBS for 1 h. For 1 h at room temperature, they were treated with a solution of NGS, TX-PBS, and specific primary antibodies. For both LH and PAG slides, this included a mouse anti-Cholera Toxin B antibody (1:1000; Abcam ab35988) as well as plus an anti-Orexin-A antibody (1:500; Abcam ab255294) for the LH slides only. Next, slides were incubated in primaries overnight (~ 18 h) at 4 °C. The following day slides were incubated at room temperature on a shaking platform for 1 h. Three TX-PBS washes, for a total of 30 min, was then completed. Afterwards slides were incubated in fluorescent secondaries for 1 h, at a dilution rate of 1:200. For both LH and PAG slides this involved a solution of TX-PBS, goat anti-rabbit Alexa Fluor 647 (ThermoFisher #A-21,245), goat anti-mouse IgG1 Alexa Fluor 488 (ThermoFisher #A-21,121) and Hoechst 33,342. Slides were protected from light exposure from this point onwards. After the incubation of fluorescent secondaries, the slides were washed with TX-PBS for 10 min, which was repeated three times.

Coverslips were mounted onto all slides with Fluoromount mounting medium (Merck F4680).

### Image acquisition, neuronal and statistical analyses

Following immunofluorescent staining, tissue sections were imaged at 20x objective using a Nikon Ti-E 100fps inverted fluorescence motorized microscope equipped with a sCMOS Andor Zyla camera lens. These sections were analysed by a researcher blinded to all experimental conditions. The tissue section that had the most dense and localised staining of the neuronal tract-tracer inferiorly was considered the centre of the injection. This tissue section, and the sections adjacent, were then analysed for the anatomical accuracy of the tracer injection. The Paxinos and Watson Rat Brain atlas [25] was used as a reference to confirm which surgeries were successful.

LH tissues sections from animals where tracer injection was deemed successful were imaged. The researcher who completed the image processing was blinded to the injury status of each animal. Image acquisition occurred with a Nikon Ti-E 100fps inverted fluorescence motorized microscope, using a sCMOS Andor Zyla camera lens. Stitched images (4 × 4) of both hemispheres of the LH were captured at 20x objective. For each brain, every third slice, for a total of 10 sections of tissue, were imaged.

Orexin and CTB cell bodies as well as BDA nerve terminals were quantified on all imaged LH sections. For each section, the left and right hemisphere were analysed separately. Image analysis for neuronal quantification was completed using Fiji (Image J) software.

### ELISAs

Two sandwich enzyme-linked immunosorbent assays (ELISAs) were conducted to examine acute changes to systemic inflammatory markers following RmTBI. Both kits; TNF-α (#RTA00, R&D Systems) and calcitonin gene related peptide (CGRP – ABIN5670712, Antibodies Online), were utilized in accordance with the manufacturer’s protocol for serum. On the 96-well plate, all standards, negative and positive controls, as well as samples were run in duplicate. Results were measured with a FLUOstar Omega microplate reader.

### Statistical analyses

Data was analysed using SPSS (27.0 for Mac). All data was analysed using one-way ANOVAs, with injury as the factor and *p* values of < 0.05 considered statistically significant. GraphPad/Prism 9 software was used to create graphs, with all graphs displaying mean ± standard error of the mean (SEM). All data can be found at the open sources framework (OSF): https://osf.io/daun9/?view_only=a4a2e89120a9490397ec423efb17db96.

## Results

### Confirmation of Injury induction and behavioural outcomes

Overall, time-to-right data confirms that RmTBI animals experienced an increased loss of consciousness thereby validating the presence of an injury (F_(1, 63)_ = 173.307, *p* < .001). In addition, the RmTBI animals exhibited a reduction in anxiety-like behaviour as indicated by increased time in the open arms (F_(1, 62)_ = 4.620, *p* = .036) and changes to thermal sensitivity on both the hot and cold plate (F_(1, 62)_ = 4.843, *p =* .032, and F_(1, 62)_ = 10.496, *p =* .002 respectively). See Fig. 1B**-**E.

### RmTBI results in an acute loss of orexin cell bodies

**Fig. 2:**
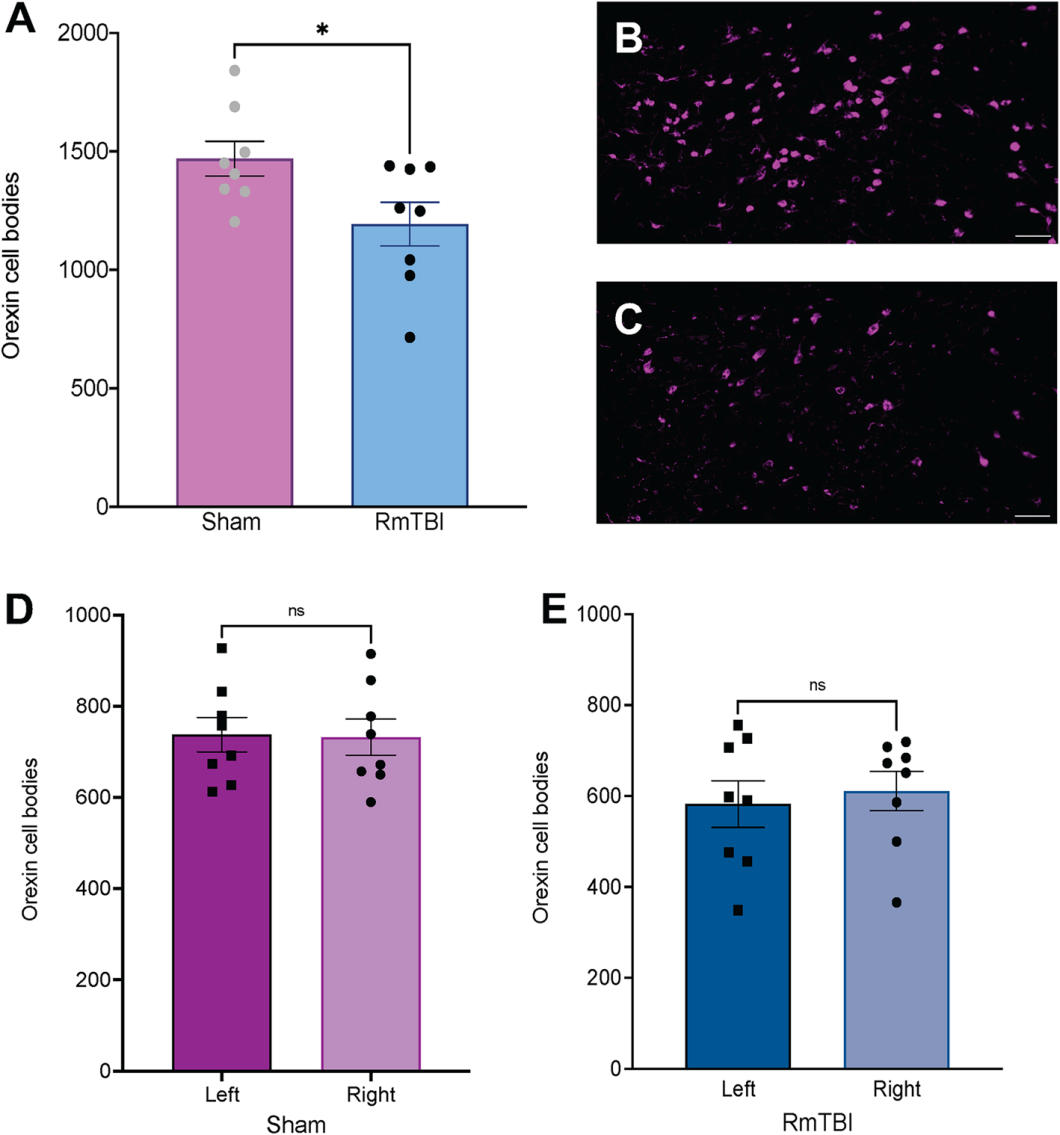
Representative images and bar graphs displaying the quantity of orexin cell bodies in the LH of sham and RmTBI animals. **A** Total number of orexin cell bodies in sham and RmTBI groups; **B)** Example image of orexin cell bodies in LH of sham animal and **C)** RmTBI animal; **D)** Number of orexin cell bodies in left and right hemisphere of LH of sham groups and **E)** RmTBI groups. Mean ± SEM shown. * *p* < .05. Scale bars represent 100µm

Brains that had successful tract-tracing surgeries were analysed for a general effect of injury on the quantity of orexin cell bodies (n = 16; 8 RmTBI and 8 Sham). The one-way ANOVA for quantity of orexin cell bodies in the LH indicated a significant effect of injury (*F*_(1,15)_ = 5.512, *p* = .034). As demonstrated in Fig. 2A, RmTBI animals displayed an overall reduction in quantity of orexin cell bodies comparative to sham animals. There was no statistically significant difference in the quantity of orexin cell bodies between the left and right hemisphere within sham animals (*F*_(1,15)_ = 1.082, *p* = .922) or RmTBI animals (*F*_(1,15)_ = 1.420, *p* = .680, see Fig. 2D-E).

### Efficacy of the tract-tracing surgeries

**Fig. 3:**
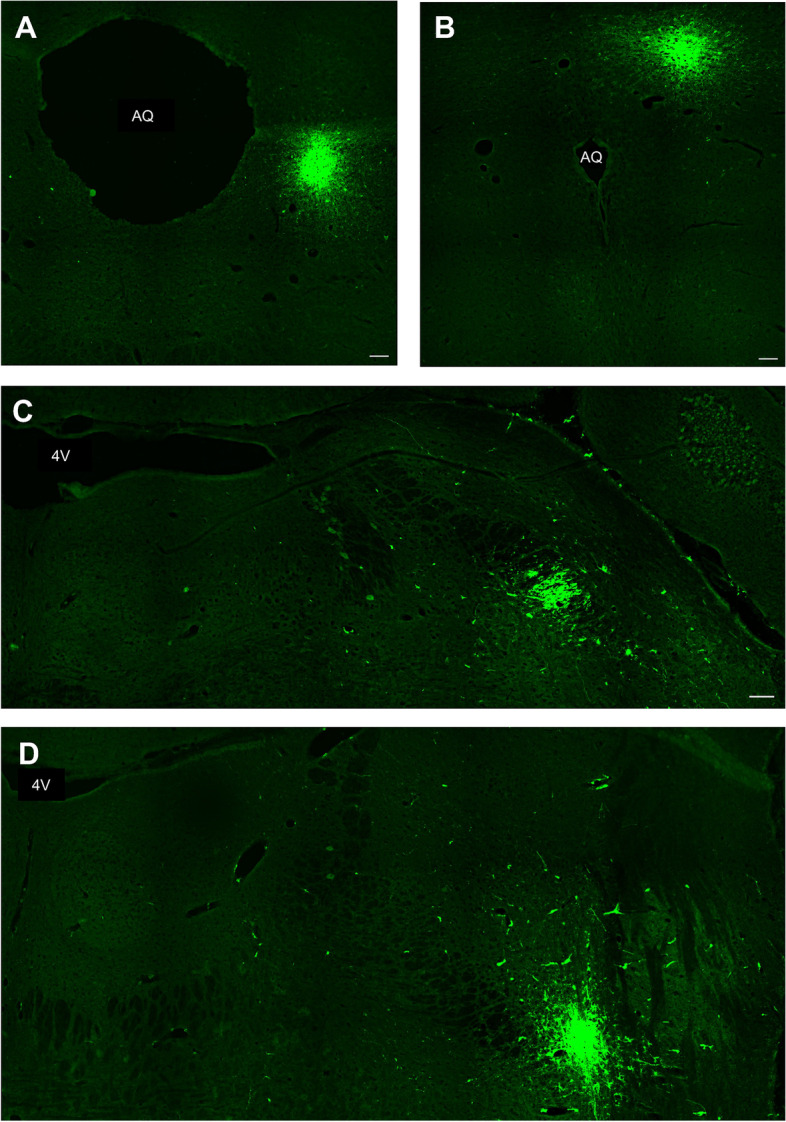
Example images of tract tracing surgery hits and misses to the PAG and lPBN. **A** An example hit to the PAG, **B**) An example of a missed injection to the PAG, **C**) Represents a successful injection to the lPBN, and **D**) Represents a missed injection to the lPBN. AQ – aqueduct, 4V – fourth ventricle. Scale bars represent 100µm

Neuronal tract-tracing surgery into the lPBN was anatomically accurate in 9/32 brains, generating a sample size of 5 RmTBI and 4 sham injured animals. Similarly, tract-tracing surgery into the PAG was anatomically accurate in 8/33, leading to a sample size of 4 RmTBI and 4 sham injured animals. Cells and nerve terminals were counted from the left and right hemispheres. As there were no significant effects of hemisphere, the analyses were collapsed. See Fig. 3 for examples of hits and misses from the tract-tracing surgeries to the lPBN and PAG.

### RmTBI did not modify connectivity between the lPBN and the LH

**Fig. 4:**
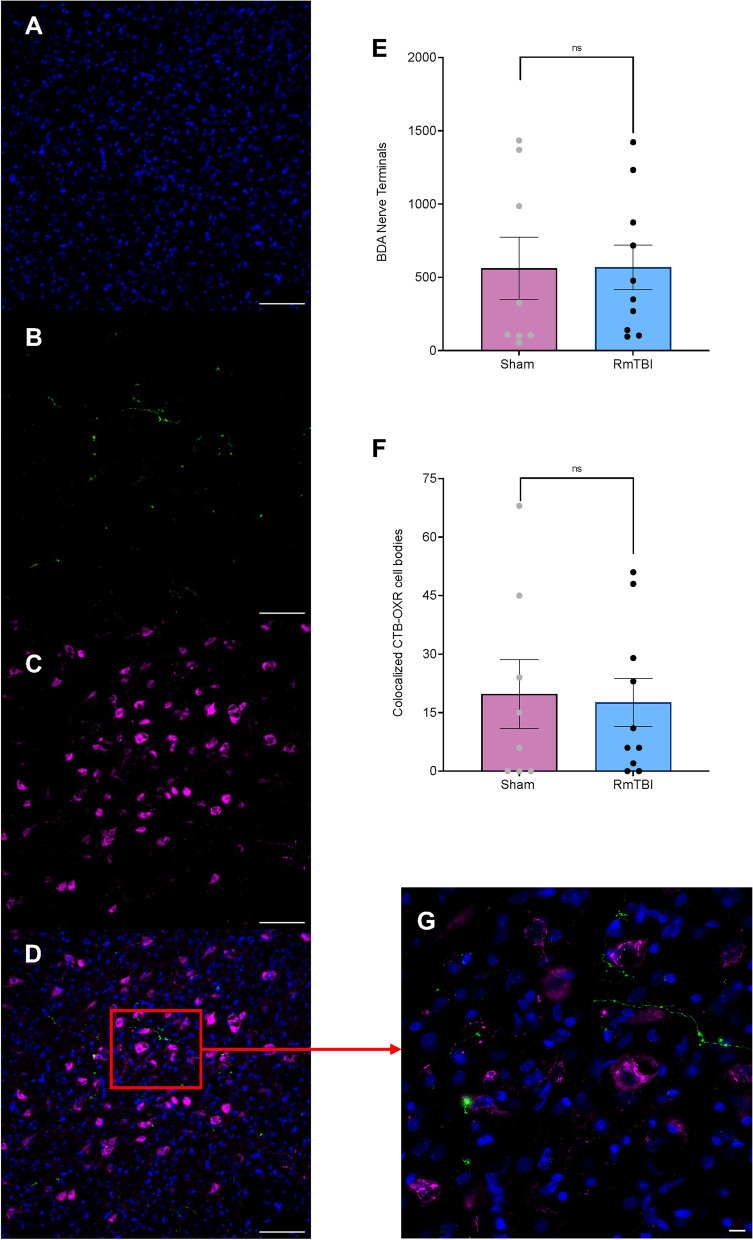
Representative images and bar graphs illustrating the quantity of BDA-labelled nerve terminals and co-localized BDA and orexin cell bodies in the LH. **A **Example image of Hoechst 33342 stained cells; **B)** Example image of BDA-labelled terminals in the LH; **C) **Example image of orexin cell bodies in the LH; **D)** Example image of co-localized BDA-labelled terminals and orexin cell bodies; BDA (green) and orexin cell bodies (magenta) - Scale bars represent 100µm; **E)** Quantification of the total number of BDA-labelled terminals in sham and RmTBI animals; **F)** Quantification of the total number of co-localized BDA-labelled terminals and orexin cells in the LH of sham and RmTBI animals; **G**) Example image, with zoom (scale 10 µm) of co-localized BDA-labelled terminals and orexin cell bodies. Mean ± SEM shown

The RmTBIs did not modify the number of projections from the lPBN to the LH. The one-way ANOVA for quantity of BDA projections did not demonstrate a significant main effect of injury, (*F*_(1,19)_ = 0.001, *p* = .978). Similarly, RmTBI did not affect the number of co-localized BDA projections and orexin cell bodies, (*F*_(1,19)_ = 0.042, *p* = .839). See Fig. 4.

### RmTBI reduced CTB cell bodies within the LH

**Fig. 5:**
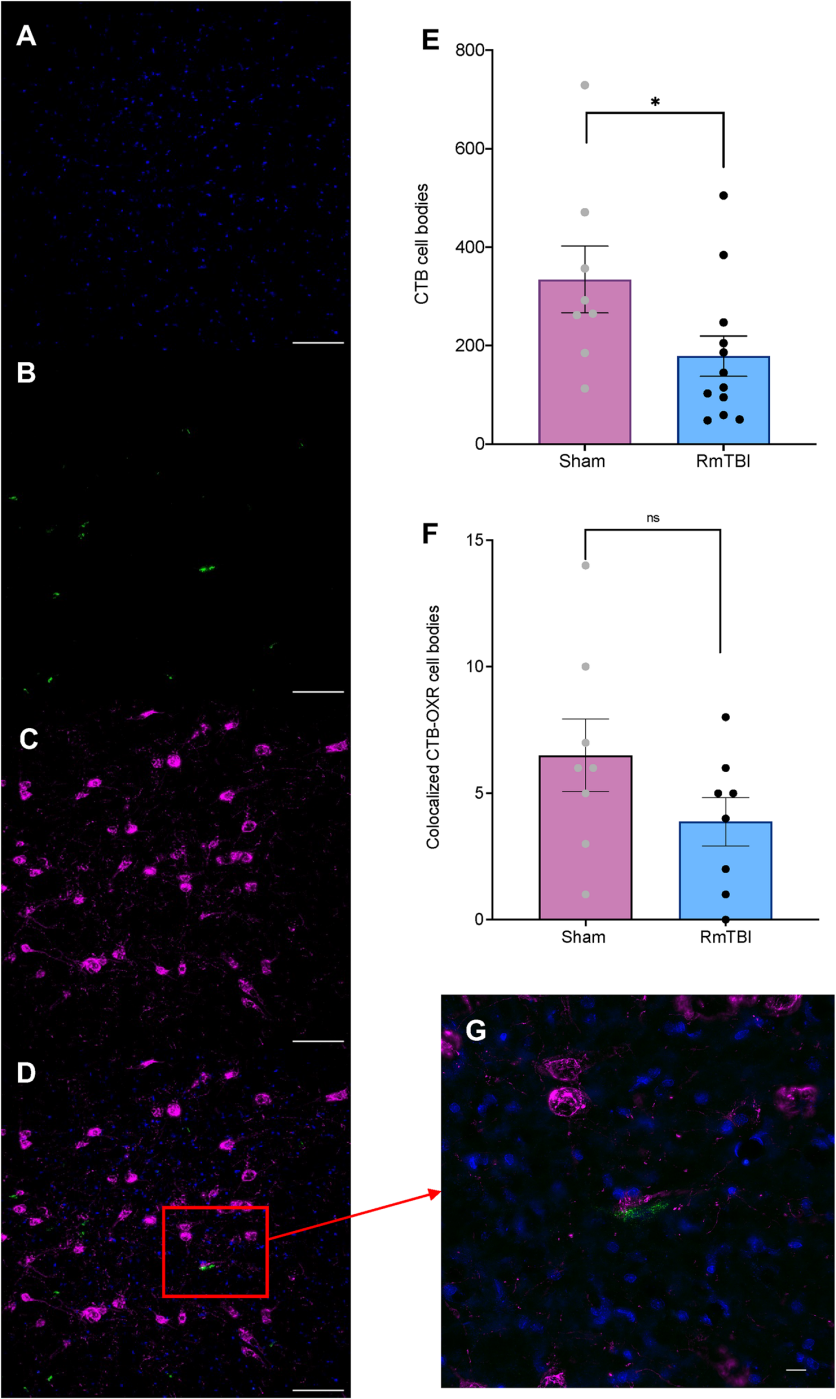
Representative images and bar graphs illustrating the quantity of CTB cell bodies and co-localized CTB and orexin cell bodies in the LH. **A **Example image of DAPI stained cells; **B)** Example image of CTB cell bodies in the LH; **C) **Example image of orexin cell bodies in the LH; **D)** Example image of co-localized CTB and orexin cell bodies; CTB (green) and orexin cell bodies (magenta) - Scale bars represent 100µm; **E)** Quantification of the total number of CTB cell bodies in sham and RmTBI animals; **F)** Quantification of the total number of co-localized CTB and orexin cells in the LH of sham and RmTBI animals; **G**) Example image, with zoom (scale 10 µm) of co-localized CTB and orexin cell bodies. Mean ± SEM shown. * *p* < .05

The one-way ANOVA analysing quantity of CTB cell bodies in the LH indicated a significant effect of injury (*F*_(1,19)_ = 4.395, *p* = .049). As demonstrated in Fig. 5, animals displayed an overall reduction in quantity of CTB cell bodies comparative to sham animals. The one-way ANOVA for co-localized CTB and orexin cells bodies failed to demonstrate a statistically significant difference between RmTBI and sham animals, (*F*_(1,15)_ = 2.340, *p* = .148).

### Changes in nociception were not associated with circulating TNF-α or CGRP

Serum levels of TNF-α were not statistically different between animals in the RmTBI group and the sham animals; (*F*_(1,37)_ = 0.552, *p* = .462). Similarly, there were no significant differences between RmTBI and sham serum levels of CGRP at 3 day post-injury, (*F*_(1,36)_ = 1.417, *p* = .244), suggesting that the identified changes in nociception likely resulted from modification to the neurocircuitry rather than systemic inflammation. Data not shown.

## Discussion

This proof-of-concept study sought to investigate the influence of RmTBIs on lPBN innervation of orexinergic neurons, orexinergic projections to the PAG, and how this may relate to pain vulnerability post-injury. RmTBI animals spent a greater amount of time in the open arms of the EPM, which may reflect impulsivity-like behaviour, rather than decreased anxiety. Altered emotional regulation in the form of ADHD-type phenotypes within males is an established occurrence post-RmTBI [33]. On the HCP we observed altered nociceptive sensitivity at an acute post-RmTBI timepoint. Injured animals showed reduced nociceptive sensitivity as indicated by a longer latency to react. We observed the novel finding, to the best of our knowledge, of a structural deficit of orexin cell bodies following RmTBI. Moreover, RmTBI also reduced orexinergic projections to the PAG, but not from the lPBN to the LH.

As anxiety is often comorbid with chronic pain, we hypothesised that injured animals would display greater anxiety-like behaviour acutely post-injury. However, contrary to our hypothesis, RmTBI animals spent an increased amount of time in the open arms of the EPM, which normally would be thought to indicate decreased anxiety-like behaviour [34–36]. It is possible that our results reflect disinhibition or impulsivity behavioural changes, which has been observed in previous research [37, 38]. For example, Mannix et al., saw persistent impulsivity in a male adolescent mouse cohort following RmTBI, indicated by increased time spent in the open arms of the EPM [39]. In rodent models, impulsivity has also been observed following a singular mTBI [40]. However, it is important to note that differences in anxiety-like behavior (ranging from reduced anxiety to increased anxiety) have been noted in various preclinical models of post-traumatic headache, particularly when studying blast-related mTBI [41].

In keeping with our hypothesis, we observed altered nociceptive sensitivity on both the hot and cold plate. Injured animals consistently demonstrated an elevated nociceptive threshold as indicated by a longer latency until behavioural indicators of nociception. This however is inconsistent with pre-clinical mTBI rodent literature, where a reduced nociceptive threshold is typically observed post-mTBI [42, 43]. Studies that have specifically utilised a thermal measure of nociception within a RmTBI cohort, observed reduced nociceptive thresholds within their injured group [27, 29]. The discrepancy between their findings and our results, could relate partly to differences in the age of rodents investigated. Both Salberg et al. [29] and Christensen et al. [27] analysed adolescent rodents while our animal cohorts were adults. It has been illustrated clinically that there is a positive correlation between an individual’s age and increased pain thresholds [44]. Additionally, the quantity of concussive head injuries has also been identified as an influential factor regarding nociceptive sensitivity [43]. We performed RmTBIs within an inter-injury interval timeframe that has been proven to have cumulative physiological effects [45], which can alter physiological homeostasis [46]. Threats to homeostasis, either physiologically or psychologically, are defined as ‘stress’ [47], and it is well documented, particularly pre-clinically, that physiological stress can induce an analgesic response upon exposure to noxious stimuli [47]. Therefore, our experimental set-up may have caused a cumulative physiological burden from five RmTBIs, across an acute time frame, and catalysed the generation of stress-induced analgesia, presenting as decreased nociceptive sensitivity.

Along with the cumulative behavioural effects associated with RmTBIs is an upregulation of cytokines and pain-related peptides, which have been observed both pre-clinically [48] and clinically [49, 50]. Interestingly, certain cytokines have been proven to have an anti-nociceptive effect by downregulating the release of pro-inflammatory cytokines [51, 52] and blocking the release of neurotransmitter in peripheral nociceptors via inhibition of calcium channels. We failed to corroborate these findings, as we did not identify changes in TNF-α in our RmTBI animals. However, this may have been a result of the timing of serum collection. Secondary signalling cascades are generally initiated and terminated acutely post-injury, and it is possible that we would have identified changes in the first 24 h [14]. Given that CGRP is a peptide found within the peripheral and central nervous system, that when elevated in blood has been linked to nociceptive transmission, the induction of headache in individuals with persistent post-concussive symptomology, as well as and migraine pain, in both humans and animals [42, 53–58], we also examined serum levels of CGRP. Contrary to our hypothesis and despite changes in nociception, RmTBI did not increase serum CGRP at two-days post-final injury in this study. Interestingly, a study by Bree et al. [59], found that CGRP was only increased 7 days post-mTBI in female rats – there were no effects in males or when serum was analyzed at 3 days. Therefore, it is possible that if sampled 7–10 days post-RmTBI our animals would have also demonstrated increased serum CGRP levels. However, consistent with Bree et al., and although negative, our results provide further evidence for the complexity of time- and sex-specific relationship that exists between mTBI, chronic pain phenotypes, and changes to neuroimmune signalling cascades.

Post-RmTBI we observed a significant reduction in the gross quantity of orexin cell bodies. To the best of our knowledge, a loss of orexin cell bodies at the level of a mild head injury is a novel finding. To date, gross orexin cellular loss has only been observed secondary to head injuries of greater severities [60, 61]. Moreover, the potential for mTBIs to generate orexinergic structural damage is currently contested [62, 63]. Three days post-mTBI, Willie et al. observed physiological alterations in the extracellular functioning of orexin neurons, but no gross deficit regarding cell count [64]. Lowered orexinergic neuronal activation has also been demonstrated post-mTBI, but again this could not be correlated with gross orexin cellular loss [63]. Therefore, the inconsistencies between our results and the literature may pertain to two key mechanisms; the method employed to induce mTBIs and the number of mTBIs administered. Previous mTBI studies that commented on gross orexin cellular loss were in the context of controlled cortical impact [64] and fluid percussion injury [63]. Less distinctive rotational forces in these models could have reduced the mechanical load, and hence shearing forces, through the orexinergic neurons, limiting the gross cellular deficit. The pathological reduction in hypothalamic volume following a severe TBI [65], and the 27% loss of orexinergic neurons post fatal TBIs comparatively [66], indicates the vulnerability of the orexinergic neurons to shearing forces [65]. Therefore, the axonal plane at which hypothalamic orexinergic neurons lie may be uniquely vulnerable to rotational mechanical loads, irrespective of severity of head injury. Secondly, RmTBIs generate a prominent state of neuroinflammation that may affect gross orexinergic cell numbers. Post-mTBI inflammation is marked by an upregulation of microglia [67], as well as an increase in astrocyte activation [68] and pro-inflammatory cytokines [69]. Orexinergic neurons have demonstrated sensitivity to such inflammatory processes, both within the CNS and peripherally. Given that the proinflammatory cytokine TNF-α reduced orexinergic function by limiting the level of its precursor prepro-orexin [70], inflammatory states may reduce the production of the orexinergic system’s neuromodulators.

An acute loss of orexinergic neurons post-RmTBI is important to the current gap in our understanding of the drivers of acute dysfunctional DPM. To reiterate, orexin has a dual anti-nociceptive influence [19]. Orexinergic neurons innervate key sites within the DPM pathway, including the PAG, alongside directly synapsing to interneurons within the dorsal horn of the spinal cord, to decrease ascending nociceptive transmission [19]. It is also important to note, that although there a restricted number of orexin neurons, they have extensive arborization with projections that are distributed throughout the brain; even a small reduction in cell number could result in significant dysfunction [13]. With respect to pain processing, a loss of orexinergic neurons would reduce the quantity of orexin binding to post-synaptic membranes, creating an imbalance in pain facilitatory and inhibitory signalling. Second, the presence of acute functional changes within the DPM, post-mTBI, is a well-established finding. The loss of descending noxious inhibitory control, a measure of descending pain modulation, has consistently been seen post-mTBI within rodent cohorts [54, 71]. Hence our results raise the possibility that physiological changes in the orexinergic system, secondary to structural losses, drive the acute deficiency in the DPM system following RmTBI.

The current findings suggest that at least acutely post-RmTBI, connectivity between the lPBN and the LH post-RmTBI, was not affected. Our results indicate that there were no changes in the number of excitatory glutamatergic projections from the lPBN to orexin cell bodies that reside in the LH. Existing literature suggests, however, that there are key physiological changes within the orexinergic system secondary to mTBIs. For example, Elliot et al. induced a mTBI in rats and discovered decreased density of glutamate within pre-synaptic connections to orexin neurons at one week post-injury [72]. Moreover, based on the presence of c-Fos expression, they determined that reduced orexin neuronal activation had been established post-mTBI [63]. Reduced excitatory input into orexinergic neurons post-mTBI would account for both a reduction in the density of glutamate to and neuronal activation of orexin neurons. Therefore, it is plausible to consider that RmTBI could induce structural changes to lPBN neurons that would, in turn, diminish its excitatory control over the orexinergic system. Such ‘structural changes’ involve the breakage of neuronal tracts, which may have interrupted the flow of BDA ions and caused a reduction in the quantity of BDA-labelled nerve terminals. This breakage, also known as, diffuse axonal injury (DAI) [73], is observed at all severities of head injuries [74]. Valko et al. found greater injury to hypothalamic neurons mediating arousal, than those derived from key brainstem sites including the midbrain (the lPBN is situated within the midbrain) [75]. In keeping with this, Ommaya et al. demonstrated that during a TBI, the brainstem is more protected from injury than other sites [76]. Therefore, the protection of the brainstem from rotational forces may limit the breakage of lPBN efferent projections and the resulting physiological effect on the orexinergic system. However, this does not negate the role of the orexinergic system in physiologically influencing mTBI outcomes but demonstrates that connectivity between the lPBN and LH may not be the driver of such physiological changes. Although the existing literature provides reasonable consideration for both the presence and absence of lPBN connectivity changes post-mTBI, our qualitative results corroborate an absence of change.

The current study demonstrated a loss of, or breakage, in hypothalamic projections to the PAG in injured animals. More specifically, animals who received five mTBIs had a reduced quantity of CTB-labelled cell bodies distributed throughout the LH, which represented output projections to the PAG. Therefore, the observed loss of hypothalamic output projections at a mild level of injury is, to the best of our knowledge, a novel finding. This result is consistent with the impact of RmTBI on orexin cell bodies in animals who received RmTBIs versus those with sham injuries (outlined above). The functional losses, likely caused by DAI, on these efferent hypothalamic projections could have a chronic implication as the specific functional changes would be dependent on the different hypothalamic nuclei affected – which have unique functional and anatomical relations [77].

Retrograde tract-tracing from the vlPAG showed that this region received innervation from the posterior LH (PLH) and the perifornical part of the LH (PeFLH). This is consistent with prior literature, where it has been extensively documented, that the primary innervation of the vlPAG is by LH [78, 79]. Similarly, orexinergic neurons have been consistently shown to originate exclusively within the LH [80]. Although we failed to demonstrate a loss of co-localized CTB and orexin cell bodies within the LH, there are still functional implications for the role of orexin within the PAG following RmTBI. For example, the binding of orexin-A to the orexin receptor within the vlPAG, increases downstream excitatory signalling to distal sites within the DPM pathway [81]. Our results indicate that there may be reduced orexinergic innervation to the vlPAG acutely post-injury, which in turn would reduce excitatory signalling within the DPM system. This expands on the discussed loss of orexin cell bodies by providing an additional mechanism that may act collaboratively to generate acute dysfunctional descending pain control. Beyond understanding why deficient DPM occurs acutely, the relevance of reduced excitatory signalling within the DPM system post-RmTBI applies to the predictive risk of acute dysfunctional DPM in the development of chronic pain. Acute maladaptive modulation of pain may predispose individuals to the chronification of pain by enabling the sensitisation of central nociceptors to noxious (and non-noxious) stimuli.

## Conclusion

This study revealed that RmTBI in young adult male rats increased impulsivity-like behaviour and nociceptive thresholds. In terms of structural changes, we demonstrated that RmTBI leads to an overall reduction in the quantity of orexinergic cell bodies and orexinergic input to the PAG, but does not influence the lPBN’s excitatory projections to orexinergic cells in the LH. Given that orexin-A has been shown to induce anti-nociception and DPM pathway activation by inhibiting the vlPAG’s intrinsic GABAergic tone [81], also known as disinhibition, this structural loss of orexinergic cells and connections to the PAG likely has significant consequences for pain processing post-injury. More specifically, after the acute period of stress-induced analgesia, it is likely that the injured group would begin to display shifts towards increased pain sensitivity, as they exhibit a deficiency in both the quantity of orexin-A and these connections that propagate its anti-nociceptive effects. Therefore, the structural damage to the orexinergic system following RmTBI may play a key mechanistic role in the development of chronic pain post-injury.

Given this novel finding, future studies should aim to examine this phenomenon at several different time points to determine how nociception and the structural integrity of the orexinergic system change at chronic timepoints. Furthermore, future investigations should determine if these structural changes are consistent for all regions of the DPM pathway that the orexinergic system exhibits modulatory control over, such as the ventral tegmental area and rostral ventromedial medulla. Studies of this nature could also employ a second immunohistochemical label for cell death to determine the time course of orexin cell loss. Given the important role of the orexins in sleep, and the known bi-directional relationship between sleep and pain/headache [82, 83], this study would have been improved if sleep duration/quality was analyzed. Importantly however, our laboratory has previously examined circadian rhythms (i.e., heart rate, body temperature, and sleep) following RmTBI with this model and found that they were largely unaffected, and only detectable when all light cues were removed and the rats were housed in total darkness [84]. Finally, it would be important to include females in future experiments, as sex could influence both the behavioural and structural outcomes.

Importantly, this is the first study, to the best of our knowledge, to expose these structural changes in the orexinergic system and its connections to the DPM pathway following RmTBI. Specifically, the present study demonstrated that a structural loss of orexinergic cell bodies and modulatory connections to regions of the DPM pathway likely contributes to acute changes in the pain response following RmTBI, and possibly the chronification of pain in the long-term. Thus, this study provides an initial avenue of insight into the structural and functional changes that occur post-mTBI and engender the development of chronic pain.

## References

[1] Cassidy D (2004). Incidence, risk factors, and prevention of mild traumatic brain injury: results from the WHO collaborating centre task force on mild traumatic brain injury. J Rehabil Med.

[2] Gardner R, Yaffe K (2015). Epidemiology of mild traumatic brain injury and neurodegenerative disease. Mol Cell Neurosci.

[3] Voss J (2015). Update on the epidemiology of Concussion/Mild traumatic brain Injury. Curr Pain Headache Rep.

[4] Thornhill S (2000). Disability in young people and adults one year after head injury: prospective cohort study. BMJ.

[5] Nampiaparampil D (2008). Prevalence of chronic pain after traumatic brain injury: a systematic review. JAMA.

[6] Lahz S, Bryant R (1996). Incidence of chronic pain following traumatic brain injury. Arch Phys Med Rehabil.

[7] Lavigne G (2015). Pain and sleep in post-concussion/mild traumatic brain injury. Pain.

[8] Uomoto J, Esselman P (1993). Traumatic brain injury and chronic pain: Differential types and rates by head injury severity. Arch Phys Med Rehabil.

[9] Lucas S (2015). Posttraumatic headache: clinical characterization and management. Curr Pain Headache Rep.

[10] Zeeberg P, Olesen J, Jensen R (2005). Efficacy of multidisciplinary treatment in a tertiary referral headache centre. Cephalagia.

[11] Benemei S (2020). Persistent post-traumatic headache: a migrainous loop or not? The preclinical evidence. J Headache Pain.

[12] Labastida-Ramirez A (2020). Persistent post-traumatic headache: a migrainous loop or not? The clinical evidence. J Headache Pain.

[13] Barkhoudarian G, Hovda D, Giza CC (2011). The molecular pathophysiology of concussive brain injury. Clin Sports Med.

[14] Giza C, Hovda D (2014). The new neurometabolic cascade of concussion. Neurosurgery.

[15] Guerriero R, GIza C, Rotenberg A (2015). Glutamate and GABA imbalance following traumatic brain injury. Curr Neurol Neurosci Rep.

[16] Ossipov M, Morimura K, Porreca F (2014) Descending pain modulation and chronificaiton of pain. 8(2):143–151 10.1097/SPC.0000000000000055 PMC4301419 24752199

[17] Ashina H (2019). Post-traumatic headache: epidemiology and pathophysiological insights. Nat Reviews Neurol.

[18] Berteotti C, Liguori C, Pace M (2021). Dysregulation of the orexin/hypocretin system is not limited to narcolepsy but has far-reaching implications for neurological disorders. Eur J Neurosci.

[19] Kang X (2021). Research progress on the mechanism of orexin in pain regulation in different brain regions. Open Life Science.

[20] Holland P, Goadsby P (2007). The hypothalamic orexinergic system: Pain and primary headaches. Headache.

[21] Arima Y, Yokota S, Fujitani M (2019). Lateral parabrachial neurons innervate orexin neurons projecting to brainstem arousal areas in the rat. Sci Rep.

[22] Niu J (2010). Glutamatergic lateral parabrachial neurons innervate orexin-containing hypothalamic neurons in the rat. Brain Res.

[23] Sun L (2020). Parabrachial nucleus circuit governs neuropathic pain-like behavior. Nat Commun.

[24] Chen J (2007). Hyperalgesia in respose to traumatic occlusion and GFAP expression in rat parabrachial nucleus: modulation with fluorocitrate. Cell Tissue Res.

[25] Paxinos G, Watson C (2013). The rat brain in stereotaxic coordinates.

[26] Mychasiuk R (2016). The direction of the acceleration and rotational forces associated with mild traumatic brain injury in rodents effect behavioural and molecular outcomes. J Neurosci Methods.

[27] Christensen J (2020). Caffeine consumption during development alters spine density and recovery from repetitive mild traumatic brain injury in young adult rats. Synapse.

[28] Eyolfson E, Genes (2021). Paternal exposure to exercise and/or caffeine and alcohol modify offspring behavioural and pathophysiological recovery from mild trauamtic brain injury in adolescence. Brain and Behavior.

[29] Salberg S (2018). A bump on the head or late to bed: the behavioral and pathophysiological effects of sleep deprivation after repetitive mild traumatic brain injury in adolescent rats. J Neurotrauma.

[30] Whishaw I, Kolb B (2005). The behavior of the laboratory rat: a handbook with tests.

[31] Le Bars D, Gozariu M, Cadden S (2001). Animal models of nociception. Pharmacol Rev.

[32] Barrot M (2012). Tests and models of nociception and pain in rodents. Neuroscience.

[33] Hehar H (2015). Impulsivity and concussion in juvenile rats: examining molecular and structural aspects of the frontostriatal pathway. PLoS ONE.

[34] Hogg S, Pharmacology (1996). A review of the validity and variability of the elevated plus maze as an animal model of anxiety. Biochem Behav.

[35] Pellow S (1985). Validation of opne: closed arm entries in an elevated plus maze as a measure of anxiety in the rat. J Neurosci Methods.

[36] Pawlak C (2012). The elevated plus maze test: Differential psychopharmacology of anxiety related behavior. Emot Rev.

[37] Collins-Praino L (2018). The effect of an acute systemic inflammatory insult on the chronic effects of a single mild traumatic brain injury. Behav Brain Res.

[38] Bortolato M (2009). Behavioral disinhibition and reduced anxiety-like behaviors in monoamine oxidase B deficient mice. Neuropsychopharmacology.

[39] Mannix R (2016). Adolescent mice demonstrate a distinct pattern of injury after repetitive mild traumatic brain injury. J Neurotrauma.

[40] Shultz S (2011). A single mild fluid percussion injury induces short-term behavioural and neuropathological changes in the Long-Evans rat: support for an animal model of concussion. Behav Brain Res.

[41] Tanaka M, Zhang Y (2022). Preclinical studies of posttraumatic headache and the potential therapeuties. Cells.

[42] Navratilova E (2019). CGRP-dependent and independent mechanisms of acute and persistent post-traumatic headache following mild traumatic brain injury in mice. Cephalagia.

[43] Tyburski A (2017). Frequent mild head injury promotes trigeminal sensitivity concomitant with microglial proliferation, astrocytosis, and increased neuropeptide levels in the trigeminal pain system. J Headache Pain.

[44] Schludermann E, Zubek J (1962). Effect of age on pain sensitivity. Percept Mot Skills.

[45] Meehan W (2012). Increasing recovery time between injuries improves cognitive outcome after repetitive mild concussive brain injuries in mice. Neurosurgery.

[46] Fehily B, Fitzgerald M (2017). Repeated mild traumatic brain injury: potential mechanisms of damage. Cell Transplant.

[47] Ahmad A, Zakaria R (2015). Pain in times of stress. Malaysian J Med Sci.

[48] Yang S (2020). Association between chronic pain and alterations in the mesolimbic dopaminergic system. Brain Sci.

[49] Chaban V (2020). Systematic inflammation persists the first year after mild traumatic brain injury: results from the prospective Trondheim mild traumatic brain injury study. J Neurotrauma.

[50] Vedantam A (2020). Early vs late profiles of inflammatory cytokines after mild traumatic brain injury and their association wiht neuropsychological outcomes. J Neurotrauma.

[51] Karam M (2011). Interleukin-13 reduces hyperalgesia and the level of interleukin-1B in BALB/c mice infected with Leishmania major with an up-regulation of interleukin-6. J Neuroimmunol.

[52] Vale M (2003). Antinociceptive effects of interleukin-4, -10, and – 13 on the writhing response in mice and zymosan-induced knee joint incapacitation in rats. J Pharmacol Exp Ther.

[53] Durham P (2006). Caclitonin gene-related peptide (CGRP) and migraine. Headache.

[54] Kopruszinski C (2021). CGRP monoclonal antibody prevents the loss of diffuse noxious inhibitory controls (DNIC) in a mouse model of post-traumatic headache. Cephalagia.

[55] Kuris A (2007). Enhanced expression of CGRP in rat trigeminal ganglion neurons during cell and organ culture. Brain Res.

[56] Mitsikostas D, Moskowitz M (2021). Making headway - a role for CGRP in post-traumatic headache. Nat Reviews Neurol.

[57] Wattiez A, Wang M, Russo A (2019). CGRP in animal models of migraine. Handb Exp Pharmacol.

[58] Ashina H (2022). CGRP-induced migraine-like headache in persistent post-traumatic headache attributed to mild traumatic brain injury. J Headache Pain.

[59] Bree D (2020). Enhanced post-traumatic headache-like behaviors and diminished contribution of peripheral CGRP in female rats following a mild closed head injury. Cephalagia.

[60] Skopin M (2015). Chronic disease in wakefulness and disruption of sleep-wake behavior after experimental traumatic brain injury. J Neurotrauma.

[61] Thomasy H (2017). Hypocretinergic and cholinergic contributions to sleep-wake disturbances in a mouse model of traumatic brain injury. Neurobiol Sleep Circadian Rhythms.

[62] Noain D (2018). Increased sleep need and reduction of tuberomammillary histamine neurons after rodent traumatic brain injury. J Neurotrauma.

[63] Lim M (2013). Dietary therapy mitigates persistent wake deficits caused by mild traumatic brain injury. Sci Transl Med.

[64] Willie J (2012). Controlled cortical impact traumatic brain injury acutely disrupts wakefulness and extracellular orexin dynamics as determined by intracerebral microdialysis in mice. J Neurotrauma.

[65] Yassin W (2015). Hypothalamic-amygdalar-brainstem volume reduction in a patient with narcolepsy secondary to diffuse axonal injury. J Clin Sleep Med.

[66] Baumann C (2009). Loss of hypocretin (orexin) neurons with traumatic brain injury. Ann Neurol.

[67] Ramlackhansingh A (2011). Inflammation after trauma: microglial activation and traumatic brain injury. Ann Neurol.

[68] Kabadi S (2014). CR8, a Novel inhibitor of CDK, limits microglial activation, astrocytosis, neuronal loss, and neurologic dysfunction after experimental traumatic brain Injury. J Cereb Blood Flow Metab.

[69] Holmin S (1997). Delayed cytokine expression in rat brain following experimental contusion. J Neurosurg.

[70] Zhan S (2011). Tumor necrosis factor-alpha regulates the hypocretin system via mRNA degradation and ubiquitination. Biochemica et Biophysica Acta.

[71] Irvine K (2018). Traumatic brain injury disrupts pain signalling in the brainstem and spinal cord. J Neurotrauma.

[72] Elliott J (2018). Dietary therapy restores glutamatergic input to orexin/hypocretin neurons after traumatic brain injury in mice. Sleep.

[73] Adams J (1989). Diffuse axonal injury in head injury: definition, diagnosis and grading. Histopathology.

[74] Fujita M, Wei E, Povlishock J (2012). Intensity- and interval-specific repetitive traumatic brain injury can evoke both axonal and microvascular damage. J Neurotrauma.

[75] Valko P (2016). Damage to arousal-promoting brainstem neurons with traumatic brain injury. Sleep.

[76] Ommaya A, Gennarelli T (1974). Cerebral concussion and traumatic unconsciousness: correlation of experimental and clinical observations on blunt head injuries. Brain.

[77] Parry D (1996). Glutamatergic projections from the rostral hypothalamus to the periaqueductal grey. NeuroReport.

[78] Semenenko F, Lumb B (1992). Projections of anterior hypothalamic neurones to the dorsal and ventral periaqueductal grey in the rat. Brain Res.

[79] Beitz A (1982). The organization of afferent projections to the midbrain periaqueductal gray of the rat. Neuroscience.

[80] Soya S, Sakurai T (2020). Evolution of orexin neuropeptide system: structure and function. Front NeuroSci.

[81] Ho Y (2011). Activation of orexin 1 receptors in the periaqueductal gray of male rats leads to antinociception via retrograde endocannabinoid (2-arachidonoylglycerol)-induced disinhibition. J Neurosci.

[82] Christensen J, Noel M, Mychasiuk R (2019). Neurobiological mechanisms underlying the sleep-pain relationship in adolescence: a review. Neurosci Biobehav Rev.

[83] Waliszewska-Prosol M (2021). Migraine and sleep - an unexplained association?. Int J Mol Sci.

[84] Yamakawa G (2019). Investigating the role of the hypothalamus in outcomes to repetitive mild traumatic brain injury: neonatal monosodium glutamate does not exacerbate deficits. Neuroscience.

